# Improving Rates of Routine Vaccinations in Adolescents at an Academic Children’s Hospital

**DOI:** 10.1097/pq9.0000000000000902

**Published:** 2026-07-28

**Authors:** Maria G. Biancarelli, Hannah Rauchle, Kathleen Waddicor, Benjamin Ethier, Sara F. Forman, Gabriela Vargas, Joshua Borus

**Affiliations:** From the * Department of Pediatrics Quality Program, Boston Children’s Hospital, Boston, Mass.; † Division of Adolescent and Young Adult Medicine, Boston Children’s Hospital, Boston, Mass.; ‡ Department of Pediatrics, Harvard Medical School, Boston, Mass.

## Abstract

**Introduction:**

This project sought to increase the vaccination completion rate to 95% in an adolescent medicine practice. We aimed to increase the percentage of individuals who were up to date on their meningococcal, human papillomavirus, and tetanus, diphtheria, and pertussis vaccinations to 80% and to verify that these efforts led to more equitable care.

**Methods:**

We used the Model for Improvement. After a current-state analysis, the team implemented a bundled intervention that included obtaining missing records, notifying providers about patients who needed vaccinations, and providing electronic medical record alerts when vaccinations were due. Patients received vaccine education, and nurses offered a numbing spray to mitigate needle-related vaccine hesitancy.

**Results:**

Statistical process control charts showed a shift in the percentage of complete vaccination records from 88% to 95%. There was a mean shift for the combined meningococcal, human papillomavirus, and tetanus, diphtheria, and pertussis completion percentage from 70% to 76%. All race and payer subgroups improved with the interventions. Racial disparity analysis for the completion percentage pre- and postintervention showed that there were 2 disparities observed between the reference group, Hispanic patients, and other racial groups. No disparities were observed between publicly and privately insured patients before or after the interventions.

**Conclusions:**

The bundled intervention was associated with higher rates of vaccine documentation and administration. The highly engaged nursing staff and innovative technology drove success.

## INTRODUCTION

Vaccinations protect against preventable, harmful diseases. Childhood vaccinations prevent 4 million deaths worldwide every year.^1^ By adolescence, most people have completed the majority of their childhood vaccine series.^2^ Both the childhood vaccination record and subsequent adolescent vaccinations are vital to care as patients enter a new medical practice.

Proper documentation of vaccine records is necessary for adolescents to attend school, work, and participate in sports. Complete vaccine records prevent unnecessary vaccine doses or laboratory titers to prove immunization.^3^ The failure of patients to bring their records to their appointments is a barrier to vaccine record completion.^4^ In our adolescent medicine practice, incomplete documentation also occurs when caregivers cannot obtain vaccination records for patients transitioning from their childhood provider or when immigrants cannot access their records. State immunization information systems provide a reliable backstop in most situations, with many states mandating their use.^5^

Meningococcal (MCV), human papillomavirus (HPV), and tetanus, diphtheria, and pertussis (Tdap) vaccines are Centers for Disease Control and Prevention–recommended and administered during adolescence and young adulthood, and are cornerstones of adolescent care.^6^ Making the most of every opportunity to vaccinate is particularly important in the current climate of rising vaccine skepticism.^7^ However, parental opposition and logistical barriers impede MCV, HPV, and Tdap vaccine administration.^8^ Specific HPV vaccine barriers include parental concern about sexual behavior, lack of school requirements, and lack of perceived benefit for those assigned male at birth.^8^ In our clinic, process-related logistics and patient acceptability were the main barriers addressed in this project.

Moreover, there are national disparities in adolescent vaccination rates. For HPV, publicly insured male Black and Hispanic patients have higher odds of being vaccinated than privately insured male White patients.^9^ Although minority groups are more likely to initiate the HPV vaccine, they are less likely to complete the whole vaccine series.^10^ This trend is not seen with other vaccines, such as the flu vaccine, where non-English speakers, Hispanic identity, public insurance, and lower income are associated with lower flu vaccination rates.^11^ This disparity has not been described in the literature for MCV and Tdap. There are several documented strategies to improve the collection of adolescent vaccination records, including reminders for providers and families, annual reviews of patients’ medical records, using every appointment as an opportunity to update vaccine records, and state immunization information systems.^3,8,12–14^ Interventions to increase vaccination rates include keeping providers up to date on vaccine recommendations, frequently offering vaccines to patients, expanding vaccination hours, and administering multiple vaccines during a single visit.^3,8,12–14^

### Specific Aims

This project, from February 1, 2022, to December 1, 2023, aimed to (1) increase the percentage of patients with a complete vaccination record from 88% to 95%; (2) increase the percentage of adolescent patients who are current with MCV, HPV, and Tdap vaccinations (from 70% to 80%); and (3) verify more equitable care. Moreover, we aimed to increase the percentage of patients up to date with MCV from 79% to 87%, HPV from 83% to 90%, and Tdap from 89% to 92% by December 1, 2023.

## METHODS

### Context

This project was conducted within the Division of Adolescent/Young Adult Medicine at a large urban academic children’s hospital in the Northeastern United States. The Division of Adolescent/Young Adult Medicine offers primary care and subspecialty programs for patients aged 12–26 years (approximately 20,434 visits/year) and sees approximately 420 new primary care patients per year. The clinic has a multidisciplinary team, including nutritionists, psychopharmacologists, mental health clinicians, including social workers and doctoral-level psychologists, and patient navigators who assist patients with social determinants of health.

### Interventions

The project primarily followed the Institute for Healthcare Improvement Model for Improvement while incorporating some Lean Management strategies.^15^ The core team included key nursing, clinical assistant, physician, and quality improvement (QI) stakeholders. Each member participated in Gemba walks to understand the current state. They interviewed key stakeholders and staff, including front desk staff and other providers. Beginning in February 2022, the team used QI tools to identify the causes of incomplete vaccine records and barriers to completing MCV, HPV, and Tdap vaccinations. The process map showed there was no standard process for gathering vaccine records when patients did not bring them to their initial appointment. (**See Supplemental Digital Content 1**, which displays the current and intervention-state adolescent immunization process map. The process map shows the initial-state vaccine record collection process and the vaccine record collection process with the intervention. We use standard process map conventions—squares indicate a task or activity, diamonds a decision point, and ovals indicate the start or finish, https://links.lww.com/PQ9/A785.) Additionally, there was no structure to prompt providers to order MCV, HPV, and Tdap vaccines for patients.

**Fig. 1.**
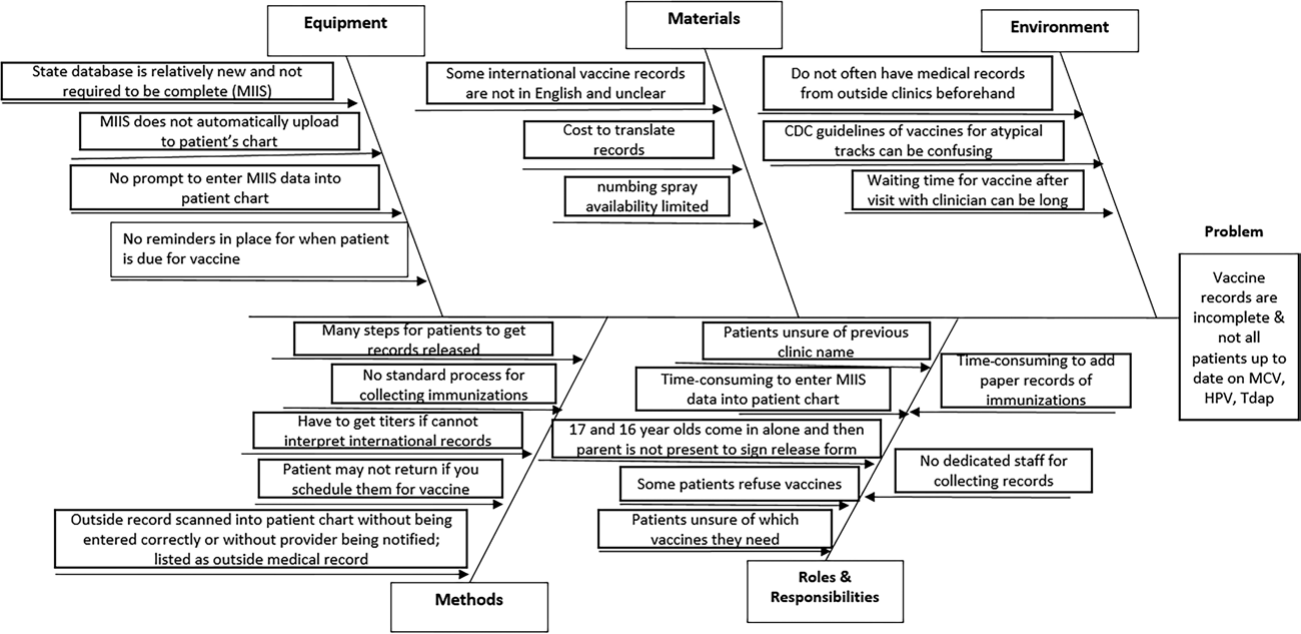
Fishbone diagram of barriers to immunization. Fishbone diagram outlining barriers to complete vaccine records and adolescent vaccination with MCV, HPV, and Tdap. CDC, Centers for Disease Control and Prevention.

**Fig. 2.**
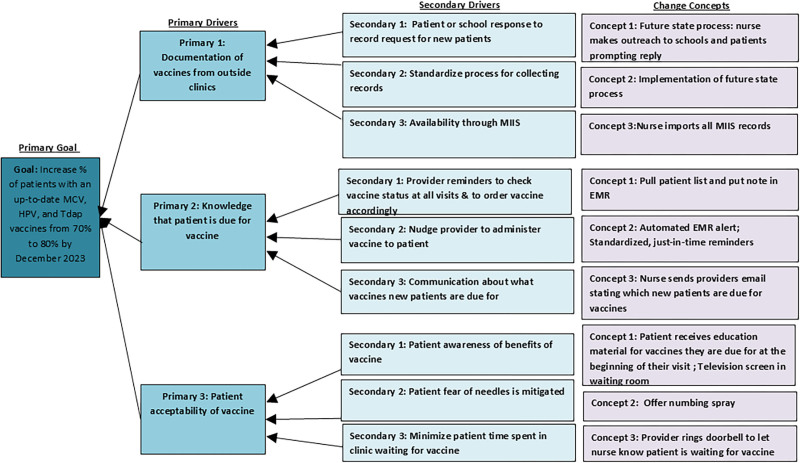
Driver diagram to increase MCV, HPV, and Tdap immunization rates. Driver diagram depicting the relationship between the aim, primary drivers, secondary drivers, and associated change strategies.

Next, the team constructed a fishbone diagram to categorize the main contributing factors to the current state of the problem (Fig. 1). The team determined that the 3 primary drivers were (1) documentation of vaccines from outside clinics, (2) knowledge that the patient is due for a vaccine, and (3) patient acceptability of vaccines (Fig. 2). The project team used evidence-based approaches aligned with the drivers to identify change concepts and applied an impact-effort matrix to prioritize interventions. The team implemented a bundled intervention approach comprising several change strategies. These were staggered in launch and consisted of 5 distinct Plan-Do-Study-Act (PDSA) cycles.

#### PDSA Cycle 1 (Tested April 11, 2022–May 5, 2022)

The first PDSA cycle targeted documentation of records from outside clinics. It created a process for nurses to import existing vaccine records from the Massachusetts Immunization Information System (MIIS). If records were unavailable or incomplete in MIIS, the nurses, with support from clinical assistants, would attempt to obtain them by contacting new patients and their families, or the patient’s previous provider or school. A flowchart depicts the process, including timelines for follow-up with nonresponding patients (**Supplemental Digital Content 1**, https://links.lww.com/PQ9/A785). The workflow underwent several iterations, revealing that this intervention was reliant on staffing.

#### PDSA Cycle 2 (Tested June 6, 2022–July 7, 2022)

The next PDSA cycle addressed provider knowledge of patient vaccine eligibility. After gathering vaccine records before patient visits, the nursing staff informed providers of new patients’ vaccination status in advance. Providers used this information to administer necessary vaccines to patients during visits. The team adopted this intervention after 1 learning cycle.

#### PDSA Cycle 3 (Tested MCV August 4, 2022–September 4, 2022, Spread to HPV and Tdap March 1, 2023)

Addressing the same primary driver, the third PDSA cycle tested semiautomated alerts in the Cerner Millennium (Oracle Corporation, Austin, TX) electronic medical record (EMR) for providers when patients were overdue for vaccinations. The internal data warehouse automatically generated a list of patients overdue for vaccines, and clinical assistants manually entered weekly alerts in the “reason for visit” bar of each patient’s EMR chart. The alert was visible to clinicians entering the chart in advance of the patient’s visit and served as a “just-in-time” reminder to offer vaccination at any visit type. The team tested this intervention on a small scale for MCV only in August 2022 and then scaled and expanded it to include HPV and Tdap in March 2023 through iterative learning cycles.

#### PDSA Cycle 4 (Tested November 7, 2022–December 7, 2022)

To address the patient acceptability of vaccines, 2 additional PDSA cycles were trialed. First, the project team reinforced the importance of patient vaccine education with clinic staff. Clinicians continued to educate patients about the importance of vaccines, and the team launched a new vaccine information sheet for patients to review during provider visits. Second, nurses and providers liberally offered patients a numbing spray to mitigate vaccine hesitancy stemming from fear of needles (PDSA 5: tested January 7, 2023–February 7, 2023). The test of change entailed nurses’ obtaining permission to stock the numbing spray and their use of scripted language with patients.

### Measures

**Fig. 3.**
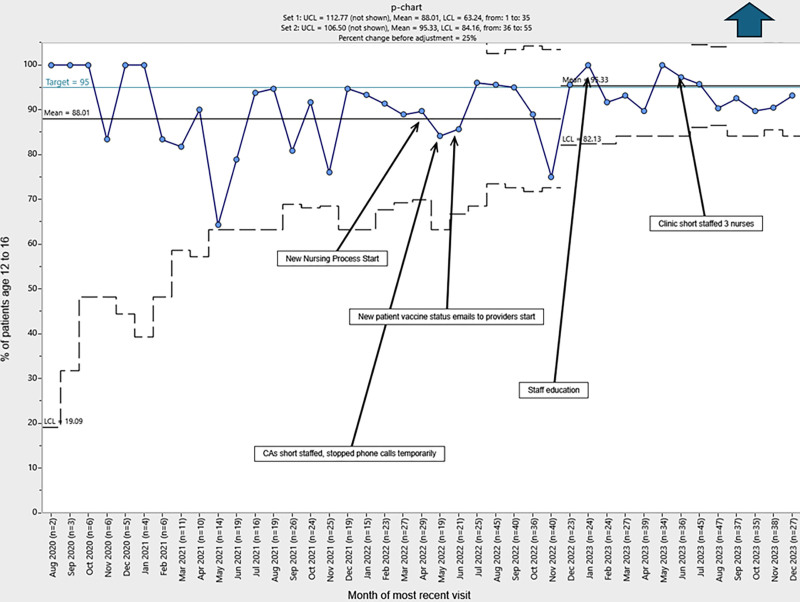
Percentage of patients aged 12–16 years with a complete immunization record. P-chart showing the percentage of patients aged 12–16 years with a complete immunization record (a proxy for > 15 vaccines). Each point represents the monthly rate. A solid black line represents the mean. The upward arrow indicates that higher is better. The dotted lines denote the statistical upper (UCL) and lower control limits (LCL). CA, Clinical Assistant.

Several measures were used to assess the effect of the intervention components. The key process measure was vaccine record completion, defined as the percentage of patients aged 12–16 years with 15 or more vaccines on record. Fifteen vaccines were used as a proxy for a complete record of the vaccines required for school, recognizing that patients may have received the standard Centers for Disease Control and Prevention vaccine schedule, the catch-up vaccine schedule, or an alternate schedule (eg, due to recent emigration). The analysis excluded seasonal vaccines (influenza and COVID-19) and HPV due to their lack of school requirements and variable lifetime dosing. Using this logic, the analysis excluded HPV from this measure, despite its acceptance as an important component of vaccination efforts (Fig. 3).

The primary outcome measure was the combined immunization completion rate for MCV, HPV, and Tdap by age 18 years. The measure counted each patient in the month of their most recent visit, ensuring that each patient was represented by a single visit. The numerator was the number of patients who by age 18 years had 1 dose of MCV after age 16 years, 2 doses of HPV, and 1 dose of Tdap or Td. Additionally, the team reported this measure separately for each vaccine type (Table 1). The team conducted a disparity analysis for the combined MCV, HPV, and Tdap measures proactively at the start of the project and again postintervention.

**Table 1.** Measurement Plans for Process, Outcome, and Balancing Measures

Measure	Denominator	Numerator
Percentage of patients aged 12–16 y with 15 or more vaccines on record (key process)	Inclusion: • Patients who had a physical examination visit in the Adolescent Medicine Clinic within the last 3 y AND • Patients aged 12–16 y AND • Patient with an in-person visit in the Adolescent Medicine Clinic in a given month - Use the patient’s most recent visit date	Inclusion: • Patients with 15 vaccines on their vaccine record at the time of their most recent visit Exclusion: • Seasonal vaccines (COVID-19, Influenza) • HPV vaccine
Percentage of patients who are vaccinated for MCV, HPV, and Tdap by age 18 y (outcome)	Inclusion: • Patients who had a physical examination visit in the Adolescent Medicine Clinic within the last 3 y AND **•** Patients had an in-person visit in the Adolescent Medicine Clinic in a given month - Use the patient’s most recent visit date	Inclusion: • Patients who received 1 dose of MCV after age 16 y and before age 18 y AND **•** Two doses of HPV AND **•** One dose of Tetanus, diptheria or Tetanus, diptheria in the past 10 y Exclusion: **•** Patients who completed vaccines after age 18 y
Percentage of patients who are vaccinated for MCV by age 18 y (stratified outcome)	Inclusion: • Patients who had a physical examination visit in the Adolescent Medicine Clinic in the last 3 y AND **•** Patients who had an in-person visit in the Adolescent Medicine Clinic in a given month - Use the patient’s most recent visit date	Inclusion: **•** Patients who received 1 dose of MCV after age 16 y and before age 18 y Exclusion: **•** Patients who completed vaccines after age 18 y
Percentage of patients who are vaccinated for HPV by age 18 y (stratified outcome)	Inclusion: • Patients who had a physical examination visit in the Adolescent Medicine Clinic in the last 3 y AND **•** Patients who had an in-person visit in the Adolescent Medicine Clinic in a given month **- **Use the patient’s most recent visit date	Inclusion: • Patients who received 2 doses of HPV by age 18 y Exclusion: • Patients who completed vaccines after age 18 y
Percentage of patients who are vaccinated for TDAP by age 18 y (stratified outcome)	Inclusion: • Patients who had a physical examination visit in the Adolescent Medicine Clinic in the last 3 y AND **•** Patients who had an in-person visit in the Adolescent Medicine Clinic in a given month - Use the patient’s most recent visit date	Inclusion: **• **Patients who received 1 dose of Tdap or Td within the past 10 y by age 18 y Exclusion: • Patients who completed vaccines after age 18 y
Count of staff hours to complete interventions (balancing)	N/A	Inclusion: **•** Number of additional hours of both clinical assistant and nursing time spent per week to complete interventions **•** Average calculated during each PDSA cycle period

The balancing measure was the number of hours the clinical assistant and nursing staff spent each week gathering vaccine records.

Analysts obtained data from our internal data warehouse, which collates EMR information. Clinicians validated all measures through chart review. The team monitored measures monthly.

### Analysis

Standard control chart rules guided the analysis of the p-charts generated with SQCPack version 7.0 (PQ Systems, Dayton, OH).^16^ The baseline period was August 2020 through March 2022, and the intervention period was until December 2023.

The disparity analysis used a retrospective cross-sectional study to compare the combined MCV, HPV, and Tdap completion rates by race and ethnicity and by payer group. Each group needed at least 30 patients. The analysis identified differences between groups by comparing each group’s performance to the reference group, defined as the best-performing group that represented at least 5% of the sample population and was not the unknown category. The analysis interpreted performance as a relative difference of at least 10% between the reference and comparison groups and a *P* value of less than 0.10, an institutional standard to highlight likely differences between groups while minimizing the chance of failing to detect a difference when one exists. This analysis examined data both pre- and postintervention. Because the control charts measured data over time and linked measurements to each patient’s most recent visit, the preintervention population for the disparity analysis differs from the baseline period group for the control charts. The team considered and measured disparities from the project’s outset, so the preintervention disparity analysis cohort comprises patients in the preperiod as of March 2022. The postintervention groups are the same for both the disparity analysis and the control charts.

This project followed local institutional standards for QI initiatives. Therefore, it was exempt from institutional review board review.

## RESULTS

**Fig. 4.**
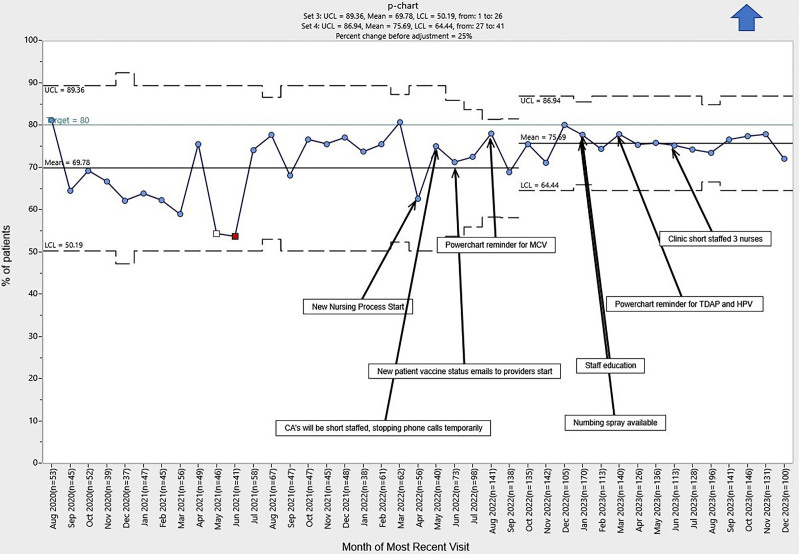
Combined completion of MCV, HPV, and Tdap. Immunizations by age 18 years. P-chart showing the percentage of patients who received the MCV, HPV, and Tdap vaccines by age 18 years. Each point represents the monthly rate. A solid black line represents the mean. The upward arrow indicates that higher is better. The dotted lines denote the statistical upper (UCL) and lower control limits (LCL).

**Fig. 5.**
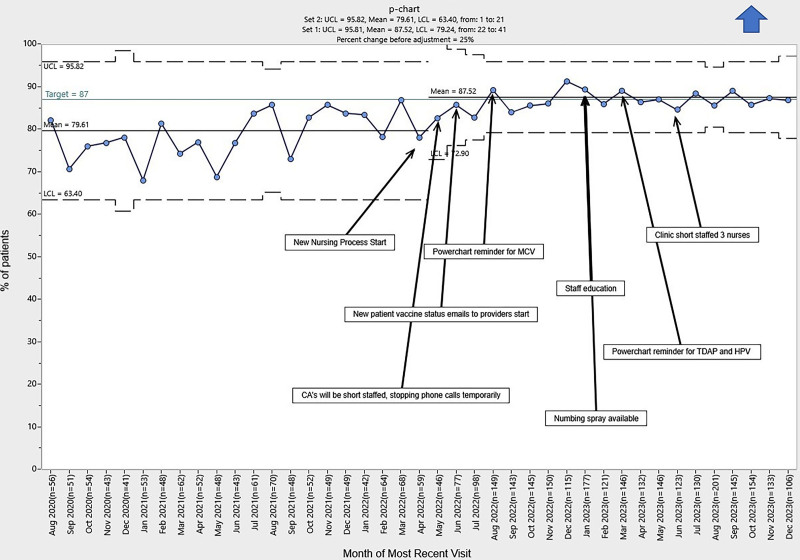
MCV vaccine completion by age 18 years. P-chart showing the percentage of patients who received the MCV vaccine by age 18 years. Each point represents the monthly rate. A solid black line represents the mean. The upward arrow indicates that higher is better. The dotted lines denote the statistical upper (UCL) and lower control limits (LCL).

**Fig. 6.**
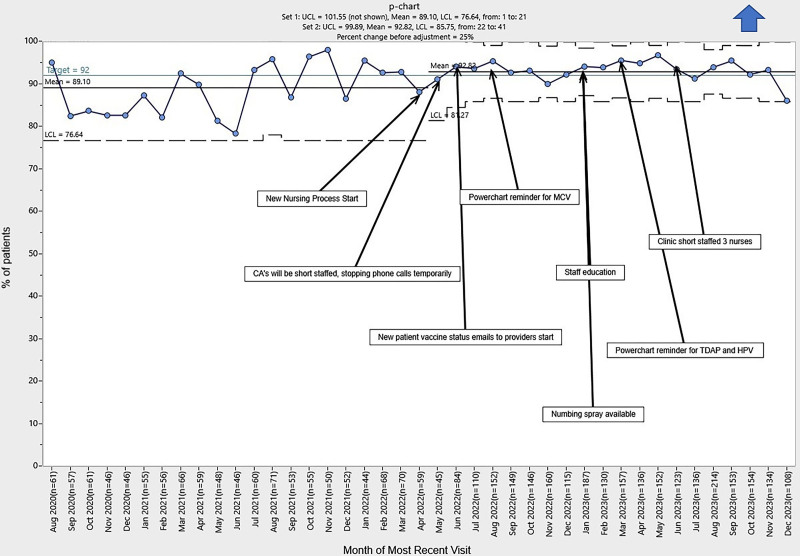
Tdap vaccine completion by age 18 years. P-chart showing the percentage of patients who received the Tdap vaccine by age 18 years. Each point represents the monthly rate. A solid black line represents the mean. The upward arrow indicates that higher is better. The dotted lines denote the statistical upper (UCL) and lower control limits (LCL).

P-chart analysis demonstrated special cause variation, with a shift in the percentage of patients aged 12–16 years with 15 or more vaccines on record from 88% at baseline to 95% beginning in December 2022, following implementation of the new process for gathering vaccine records (Fig. 3). Similarly, there was special cause variation in the combined MCV, HPV, and Tdap completion percentage, with a mean shift from 70% to 76% once several interventions were underway, including the semiautomated MCV reminder (Fig. 4). Upon initiation of the new process, there was special cause variation, with the MCV immunization completion percentage increasing from 80% to 87% (Fig. 5). For the HPV immunization completion percentage, the mean remained at 83% throughout the baseline and project periods. (**See Supplemental Digital Content 2**, which displays HPV vaccine completion rate by age 18 y. P-chart showing the percentage of patients who received the MCV immunization by age 18 y. Each point represents the monthly rate. A solid black line represents the mean. The upward arrow indicates that higher is better. The dotted lines denote the statistical upper and lower control limits. LCL, lower control limit; UCL, upper control limit, https://links.lww.com/PQ9/A786.) The Tdap immunization percentage increased from 89% to 93%, coinciding with the launch of the first intervention (Fig. 6). Notably, there is also evidence of early change before the implementation, during the planning stages of the work, indicating the Hawthorne effect.

There were 2,341 patients during the intervention period for the combined MCV, HPV, and Tdap outcome measure. The average age of patients was 20.3 years (SD = 2.8 y). Most patients were female (62%), English-speaking (88%), and had public insurance (56%). The most common race and ethnicity identification in our population was Black, non-Hispanic (38%), followed by Hispanic (23%). (**See Supplemental Digital Content 3**, which displays the demographic characteristics of primary care patients during the intervention period: March 2022–December 2023. Table reporting patients’ sex, race and ethnicity, primary language, and insurance type of patients in population, https://links.lww.com/PQ9/A787.)

Racial disparity analysis for the combined MCV, HPV, and Tdap measure showed that preintervention (before March 2022), the highest performing group was Hispanic patients (61% completion), with 2 disparities observed between this reference group and other racial groups. The disparate groups were White, non-Hispanic (53% completion, −14% relative difference, *P* = 0.037), and those of unknown race (49% completion, −19% relative difference, *P* = 0.012). After the intervention began (March 2022–December 2023), the reference group consisted of Hispanic patients (81% completion), with 2 disparities noted. These were White, non-Hispanic (66% completion, −17% relative difference, *P* < 0.001) and unknown race (72% completion, −10% relative difference, *P* < 0.001). Our largest racial and ethnic group, Black, non-Hispanic patients, improved from 57% at baseline to 76% following interventions. Within this race, the population as a whole showed a statistically significant postintervention improvement compared with baseline (57%–76% completion, +33%, *P* < 0.001). All subgroups showed an improvement of at least 24% compared with the preintervention period. No disparities emerged between publicly and privately insured patients before or after interventions. Within the payer groups, there was a statistically significant improvement in publicly insured patients (+36%, *P* = 0.051) and privately insured patients (+23%, *P* = 0.074) after interventions began, compared with the preintervention period.

The balancing measure indicated that the intervention required an average of 4 additional staff-hours per week to complete. This consisted of 3 hours of nursing time and 1 hour of clinical assistant time.

## DISCUSSION

### Summary

Using a bundled intervention strategy, the percentage of complete vaccine records improved from 88% to 95%. The combined MCV, HPV, and Tdap immunization percentage improved from 70% to 76%, missing the 80% target but sustaining improvement. Individually, completion percentages for MCV improved from 79% to 87%, and for Tdap from 89% to 93%. Interestingly, there were no statistically significant improvements in HPV completion percentages, suggesting that additional patient-facing educational interventions are warranted, given the contrast with improvements in MCV and Tdap, which were driven by process changes. The equity analysis showed improvements across all racial groups, indicating equitable distribution of the intervention’s impacts. By payer, both publicly and privately insured patients improved, with no disparities observed. The balancing measure showed that although an additional 4 hours per week of staff time was needed, this did not impose an inordinate burden on clinical operations.

The project has several strengths. The highly engaged nursing staff was a main driver of success. Nurses understood the importance of vaccine records, and structures and resources were put in place to enable nurses to use MIIS or to connect with families, schools, or previous clinics. The second driver of success was the EMR alert. These “just-in-time” reminders for providers served as a transformative intervention, enabling patients to receive vaccines at every visit. These strategies have been noted in other work.^17^

### Interpretation

The bundled intervention approach, including patient outreach, patient education, and technology fixes, was associated with increases in vaccine documentation and administration rates. The individualized outreach, coupled with technological reminders, enabled equitable improvements in care across racial groups and maintained equity among payer groups. After the current-state analysis, the practice successfully addressed barriers to obtaining vaccination records and to administering vaccines.

Regarding vaccine documentation, the approach did not make the patient or caregiver solely responsible for maintaining their vaccine record. Staff contacting patients’ schools or prior clinics eased the process. This intervention aligns with recommendations to tailor practices to the community served.^14^ One of the greatest barriers to adolescent vaccine delivery during clinical visits is the consistency and urgency of provider vaccine reminders.^18^ The “just-in-time” reminder intervention enabled providers to consider patient vaccine status at every visit. Furthermore, previous work showed that an EMR improved HPV vaccination rates among male patients at a student health center.^19^ This project expands on this intervention by creating an alert for all recommended adolescent vaccines. To improve patient accessibility, offering a numbing spray was a low-cost, high-impact, low-effort intervention that reduced fear of pain.^20^

The analysis showed that care remained equitable for payer subgroups. Given the established racial disparities in annual health maintenance examinations,^21^ our intervention normalized vaccination rates and addressed potential inequities across all clinic visits. Although there were improvements among all racial and ethnic groups, disparities persisted between the reference group, Hispanic patients, and patients who are White non-Hispanic, as well as those of unknown race. Further exploration of persistent inequities to create tailored interventions is the next step.

Improvement remained stable during the active intervention period. After December 2023, the data were in the monitoring mode, and the mean shifts persisted through May 2024, at which point our institution switched EMR systems to Epic Systems Corporation (Verona, WI), significantly impacting data capture. For sustainment, the new EMR system will support fully automated alerts. The new process, coupled with EMR reminders, helped integrate timely vaccine documentation and administration into the practice’s culture. It improved patient care without introducing unnecessary constraints on clinic flow. Moreover, it reduced costs for the healthcare system as Tdap and MCV vaccination rates increased.^22^ Improved vaccination record completion resulted in fewer titers or repeat vaccinations.

### Limitations

The initiative has limitations. Results may not be generalizable, as this project took place at a single practice and may be challenging to spread at a network level.^23^ It addresses routine vaccinations and not “seasonal” vaccines, which may require other strategies.^24^ Additionally, the alert’s semiautomated design limited updates to once per week and therefore did not capture patients who scheduled an appointment for the same week after Monday. Another limitation is that part of the process relies on staff resources, so future success may depend on consistent staffing. Additionally, because multiple analytical methods were used, the reported baseline reflects the most conservative measure of improvement, and more patients likely benefited than were captured by the control chart. Consequently, the disparity analysis reported a 57% preintervention baseline, whereas the control chart reported a 70% baseline for the combined vaccine administration measure. Additionally, the vaccine record measure is imperfect, as we selected the most common combination of vaccines that a patient would receive by age 12 years. As part of a QI project, it was important to establish a baseline against which to measure progress. Finally, the intervention did not differentiate between missed vaccines due to clinicians not offering them and those due to patients declining them.

## CONCLUSIONS

The bundled intervention approach improved adolescent vaccine record collection and administration rates. The semiautomated alert and individualized efforts by nursing staff boosted performance. Next steps include fully automating the EMR alert and ongoing monitoring of racial disparities within the population.

## Supplementary Material
