# Supplementary figures and images for: Improving Rates of Routine Vaccinations in Adolescents at an Academic Children’s Hospital
Source: Pediatr Qual Saf. 2026 Jul 28;11(4):e902. doi: 10.1097/pq9.0000000000000902 (PMC13412655; doi:10.1097/pq9.0000000000000902)

Current State- Vaccine Record Collection

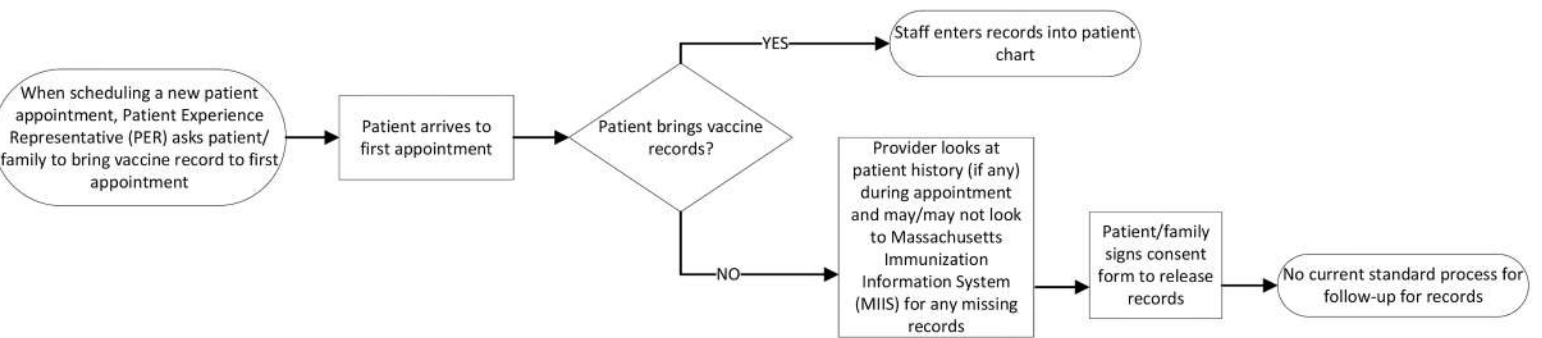

Intervention State- Vaccine Record Collection

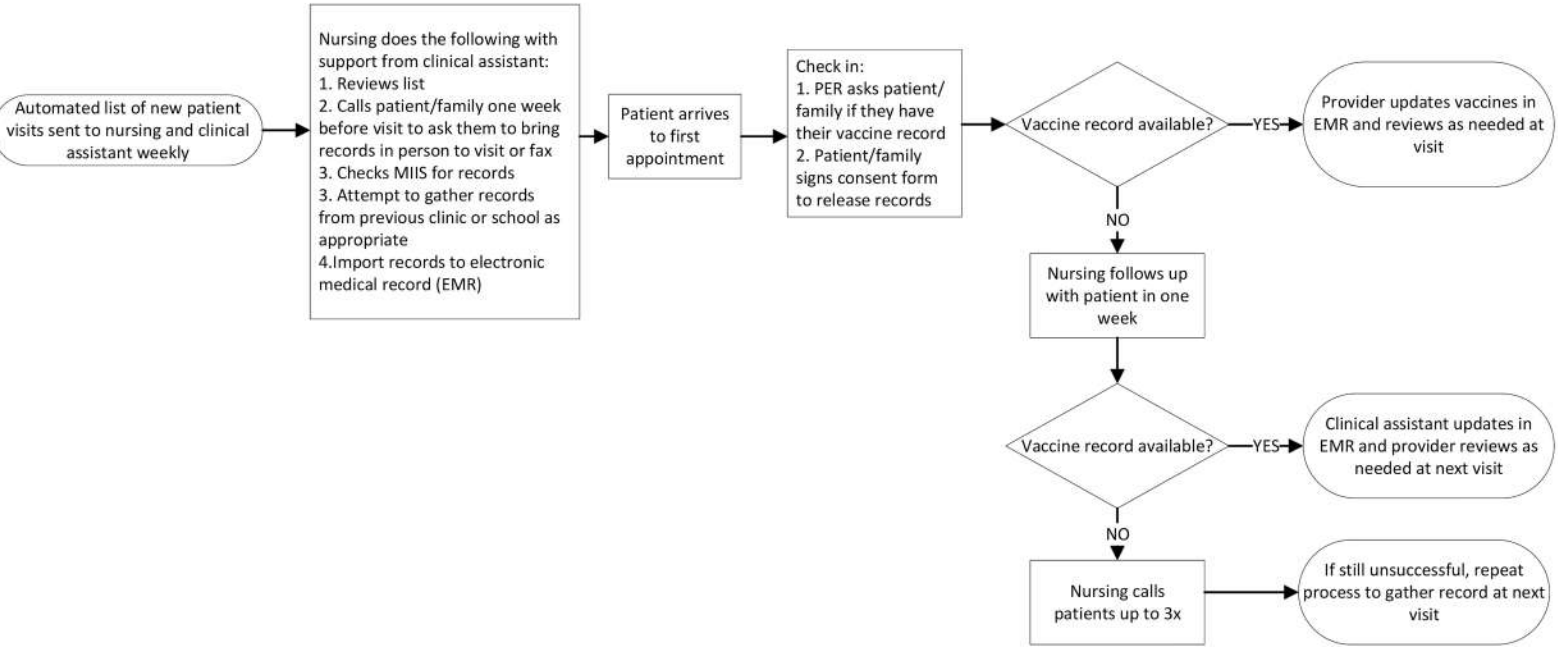

Supplement: Supplementary file 1 [file pqs-11-e902-s001.pdf]

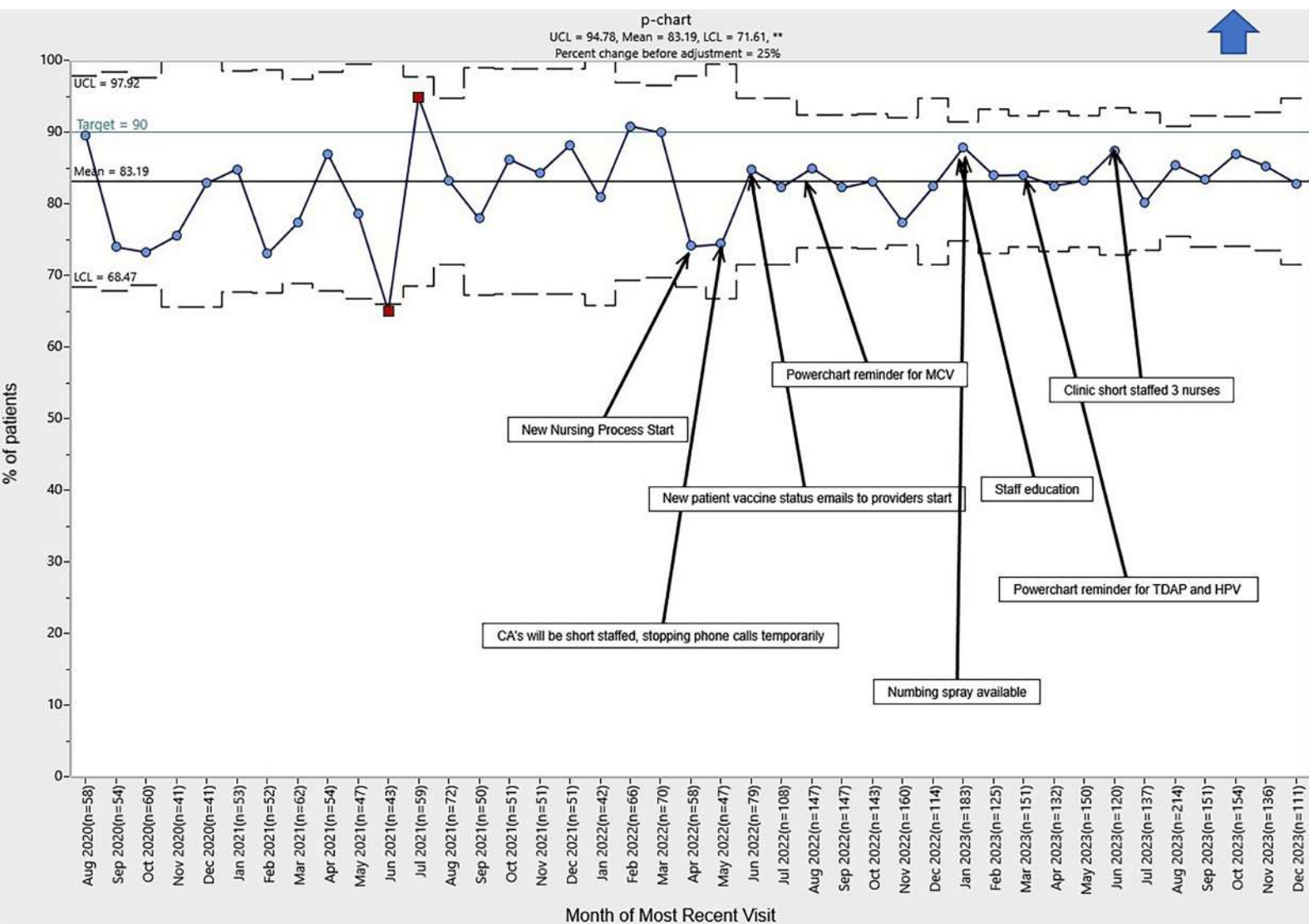

Supplement: Supplementary file 2 [file pqs-11-e902-s002.pdf]
