# Supplementary material for: Improving Rates of Routine Vaccinations in Adolescents at an Academic Children’s Hospital
Source: Pediatr Qual Saf. 2026 Jul 28;11(4):e902. doi: 10.1097/pq9.0000000000000902 (PMC13412655; doi:10.1097/pq9.0000000000000902)
Supplement: Supplementary file 3 [file pqs-11-e902-s003.pdf]

**Demographic Characteristics of Primary Care Patients During the Intervention Period: Between March 2022- December 2023**		
	**Total (N)**	**Percentage**
**All Subjects**	2,341	100
**Sex**		
Male	883	37.72
Female	1457	62.24
Other	1	0.04
**Race & Ethnicity**		
Another Race, non-Hispanic	235	10.04
Asian, non-Hispanic	84	3.59
Black, non-Hispanic	901	38.49
Hispanic	540	23.07
Multiracial, non-Hispanic	46	1.96
White, non-Hispanic	390	16.66
Unknown	145	6.19
**Primary Language**		
English	2059	87.95
Non-English	282	12.05
**Insurance Type**		
Private	970	41.44
Public	1311	56.00
Not Reported	60	2.56
